# Assessment of Redundant Meta-Analyses on Catheter Ablation of Atrial Fibrillation in Patients with Heart Failure

**DOI:** 10.31083/j.rcm2511418

**Published:** 2024-11-21

**Authors:** Luxiang Shang, Mengjiao Shao, Mingqi Zhu, Jie Li, Mei Gao, Yinglong Hou

**Affiliations:** ^1^Department of Cardiology, The First Affiliated Hospital of Shandong First Medical University & Shandong Provincial Qianfoshan Hospital, 250014 Jinan, Shandong, China; ^2^Medical Science and Technology Innovation Center, Shandong First Medical University & Shandong Academy of Medical Sciences, 250062 Jinan, Shandong, China; ^3^The Fifth Affiliated Hospital of Sun Yat-sen University, 519099 Zhuhai, Guangdong, China; ^4^Shandong First Medical University & Shandong Academy of Medical Sciences, 250062 Jinan, Shandong, China

**Keywords:** redundant publications, meta-analysis, heart failure, atrial fibrillation, catheter ablation

## Abstract

**Background:**

The utilization of catheter ablation among patients with atrial fibrillation (AF) and heart failure (HF) has garnered significant attention. There has been a rapid proliferation of diverse articles addressing this topic. This study evaluated the potential redundancy in meta-analyses about this subject.

**Methods:**

We searched PubMed, Embase, and the Web of Science for meta-analyses comparing catheter ablation with other therapies among patients with AF and HF from the inception date to December 25, 2023. The extracted data encompassed details about the author, country, publication time, journal, pre-registration status, number and type of included studies, primary endpoints, and results. Additionally, we scrutinized whether these meta-analyses referenced, described, or discussed prior relevant meta-analyses, or were cited within prominent international guidelines.

**Results:**

A total of 34 meta-analyses were included. Authors predominantly originated from the United States and China. The majority of articles were published in cardiovascular journals without pre-registration. There were two publication peaks, notably in 2018–2019 and 2023. Primary endpoints predominantly focused on all-cause mortality and alterations in left ventricular ejection fraction (LVEF). A consistent trend emerged across most articles, indicating a 40–50% reduction in mortality and a 5–9% elevation in LVEF associated with catheter ablation. Approximately 79.4%, 64.7%, and 50% of the articles respectively cited, described, and discussed previous meta-analyses on the same subject. Only 9 meta-analyses were referenced in impact international guidelines.

**Conclusions:**

Our study demonstrates a notable prevalence of redundant meta-analyses within the domain of catheter ablation among patients with AF and HF.

## 1. Introduction

Meta-analysis, a method consolidating and statistically analyzing data from diverse independent studies, offers comprehensive insights into specific topics. High-quality meta-analyses, particularly those grounded in randomized controlled trials (RCTs), serve as pivotal evidence for medical guidelines and clinical decision-making. Moderate meta-analyses, conducted at appropriate intervals to ensure relevance and avoid redundancy, are necessary as they can integrate the latest evidence, increase statistical power, resolve deficiencies in previous meta-analyses, and also help avoid repetitive RCTs [1].

Over the last few decades, the number of biomedical papers published has surged, paralleled by a remarkable upswing in meta-analyses publications. A recent study showed that the number of published meta-analyses increased by nearly 27-fold from 1994 to 2014 [2]. Nevertheless, an alarming trend of redundant meta-analyses has emerged in genetics, oncology, dermatology, and other fields [3, 4, 5]. Notably, the volume of published meta-analyses has eclipsed that of original studies. These redundant meta-analyses not only foster repetition but also sow confusion and controversy with conflicting results, impeding medical science’s advancement [2].

Atrial fibrillation (AF) and heart failure (HF) coexist in nearly 30% of patients and correlate with unfavorable prognoses [6]. While the optimal treatment for these patients remains unclear, catheter ablation has surfaced as a promising therapeutic avenue. Studies evaluating the efficacy and safety of catheter ablation for AF in patients with HF have increased markedly. This study aims to assess the redundancy prevalent in meta-analyses within this domain.

## 2. Methods

In this study, we selected eligible meta-analyses comparing catheter ablation with other therapies among patients with AF and HF. We followed the principles of the Preferred Reporting Items for Systematic Reviews and Meta-Analyses (PRISMA) guidelines [7] and the PRISMA Checklist 2020 was used for quality checking of the systematic review. This study did not involve any patient data and was therefore exempt from informed consent and ethics review. No prior registration was conducted for this study.

### 2.1 Literature Search

A comprehensive online search was conducted in PubMed, Embase, and the Web of Science databases. Detailed search strategies for these three databases are outlined in **Supplementary Table 1**. Key search terms included “atrial fibrillation”, “heart failure”, “catheter ablation”, and “meta-analysis”. Two investigators (LXS and MQZ) independently identified relevant studies from the inception of databases to December 25, 2023.

### 2.2 Study Selection 

The criteria for inclusion of meta-analyses in this study were as follows: (1) The study population comprised patients with AF and HF, regardless of the type and etiologies of HF; (2) Comparison of catheter ablation with other treatment modalities, encompassing drugs, devices, etc.; (3) Reporting of cardiovascular related outcomes; (4) Inclusion of meta-analyses based on RCTs, with potential inclusion of meta-analyses incorporating both RCTs and observational studies. We excluded original studies, case reports, reviews, editorials, letters, animal experiments, and all non-full-length publications.

### 2.3 Data Collection and Results Display of Included Meta-Analyses

Two authors (LXS and MJS) independently extracted information from each eligible study. Any discrepancies were deliberated and resolved through consensus in a meeting involving a third investigator (MQZ). Data on the first author, journal name, study location, publication date, online-search date, numbers of enrolled RCTs, study outcomes, type of pooled effect, and type of analysis were systematically recorded using a pre-designed electronic form. The original RCTs incorporated by meta-analyses were also recorded. Forest plots were utilized to visually represent the primary study outcomes of the included meta-analyses, including effect sizes and 95% confidence intervals. As our study aims to investigate the current status of redundant meta-analyses, we did not conduct assessments for risk of bias, heterogeneity evaluation, syntheses of results, or sensitivity analyses.

### 2.4 Citations and Cited Analysis of Included Meta-Analyses

Each meta-analysis article included in the study underwent a meticulous review where citations, descriptions, and discussions of previously published meta-analyses on this topic were recorded. The delineations of citation, description, and discussion were established in line with the framework introduced by Helfer *et al*. [8]. In short, citation refers to instances where the article references any previously published meta-analysis on the topic, description entails presenting the results or conclusions of prior meta-analyses within the text, while discussion involves a comparative or analytical evaluation of those preceding findings.

Moreover, the cases in which the included meta-analyses were cited within renowned guidelines on AF or HF were meticulously documented. The relevant guidelines referenced here were issued by the European Society of Cardiology (ESC), the American College of Cardiology (ACC), or the American Heart Association (AHA).

## 3. Results

### 3.1 Characteristics and General Information of the Included Meta-Analyses

**Fig. 1.**
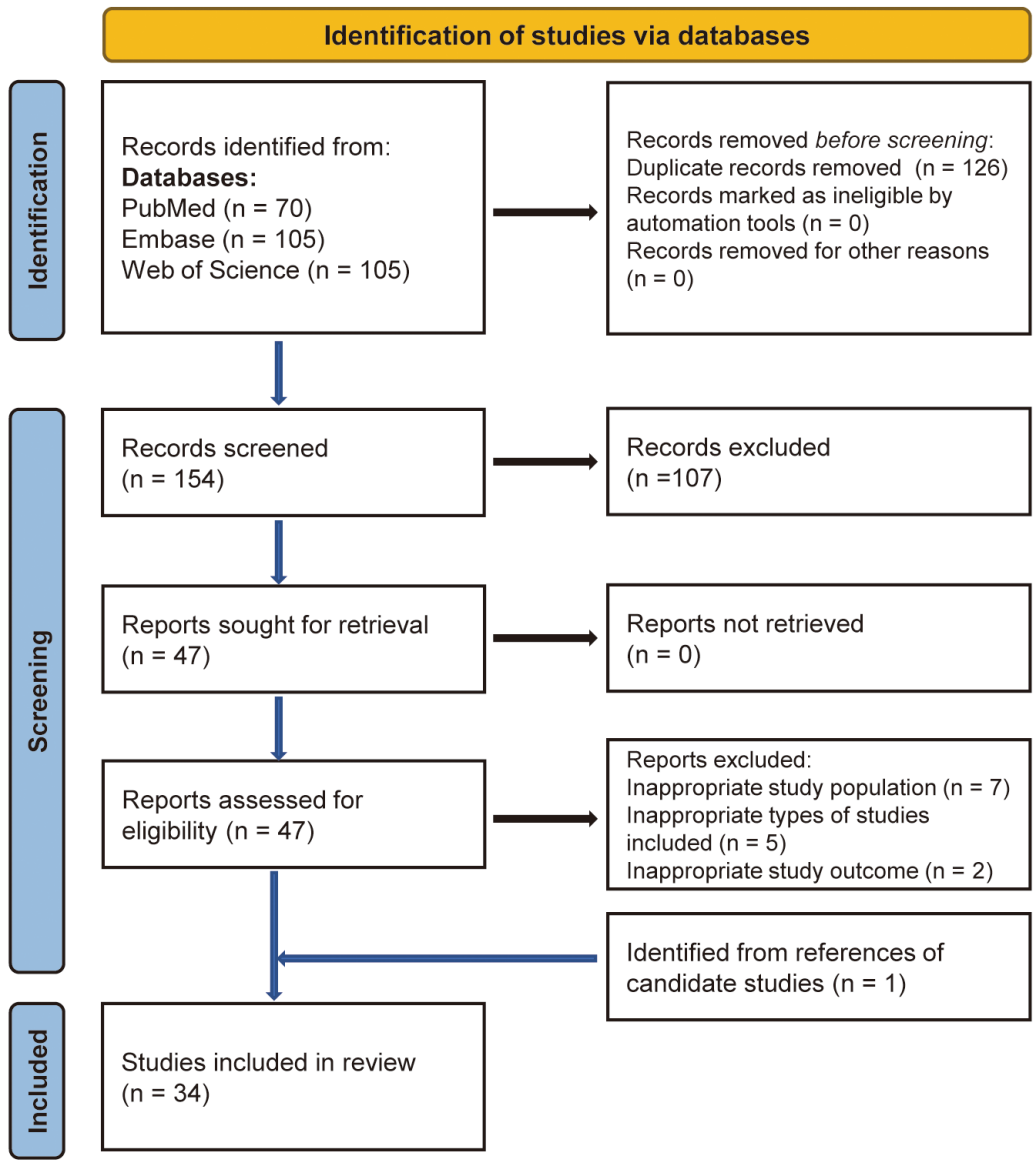
**Flow diagram for literature search and identification**.

All articles underwent screening following the PRISMA 2020 flow chart (Fig. 1). After eliminating duplicates and records failing to meet inclusion criteria, and cross-referencing potential articles, a total of 34 meta-analyses were included in the study [9, 10, 11, 12, 13, 14, 15, 16, 17, 18, 19, 20, 21, 22, 23, 24, 25, 26, 27, 28, 29, 30, 31, 32, 33, 34, 35, 36, 37, 38, 39, 40, 41, 42]. Table 1, Table 2 (Ref. [9, 10, 11, 12, 13, 14, 15, 16, 17, 18, 19, 20, 21, 22, 23, 24, 25, 26, 27, 28, 29, 30, 31, 32, 33, 34, 35, 36, 37, 38, 39, 40, 41, 42]) present the characteristics and general information of these included meta-analyses. Predominantly, articles hailed from authors based in the United States (12/34, 35.3%) and China (9/34, 26.5%). The majority of these articles found their place in cardiovascular professional journals (28/34, 82.4%), with only 2 articles appearing in high-impact journals (2022 Impact Factor >10), and most lacking pre-registration (28/34, 82.4%). Regarding publication timeline, the earliest meta-analysis dates back to 2011, with publication peaking notably in 2018–2019 and 2023. Early meta-analyses (pre-2018) primarily focused on changes in left ventricular ejection fraction (LVEF), while later ones predominantly centered around hard endpoints (such as all-cause mortality). All meta-analyses were conducted at the study level, none at patient level.

**Table 1.** **Characteristics of included meta-analyses**.

Characteristics		N (%)
Location of the corresponding author		
	USA	12 (35.3)
	China	9 (26.5)
	Australia	3 (8.8)
	Italy	2 (5.9)
	UK	2 (5.9)
	Other	6 (17.6)
Type of the journals		
	Cardiovascular professional journals	28 (82.4)
	General journals	6 (17.6)
Impact Factor of journals*		
	<5	28 (82.4)
	5–10	4 (11.8)
	>10	2 (5.9)
Year of publication		
	2011	1 (2.9)
	2015	2 (5.9)
	2016	2 (5.9)
	2018	8 (23.5)
	2019	5 (14.7)
	2020	2 (5.9)
	2021	3 (8.8)
	2022	3 (8.8)
	2023	8 (23.5)
Pre-registered		
	Yes	6 (17.6)
	No	28 (82.4)
Type of included studies		
	Mixed (RCTs + cohorts)	3 (8.8)
	RCTs only	31 (91.2)

Note: *2022 Impact Factor from Clarivate Analytics. RCTs, randomized controlled trials.

**Table 2.** ** General information of the included meta-analyses**.

Authors	Pre-registered	Publication date	Online search date	Number of included studies	Type of included studies	Primary/Main outcomes	Pooled effect	Type of analysis	Type of data
Dagres *et al*. [9]	No	2011.11	2011.04	9	Mixed (RCTs + cohorts)	Change of LVEF	MD	Random effect	Study level
Vaidya *et al*. [10]	No	2015.08	2014.04	7	RCTs only	Change of LVEF	WMD	Random effect	Study level
Al Halabi *et al*. [11]	No	2015.06	2015.02	4	RCTs only	Change of LVEF	MD	Random effect	Study level
Zhang *et al*. [12]	No	2016.06	2014.06	6	Mixed (RCTs + cohorts)	Change of LVEF	WMD	Fixed and Random effect	Study level
Zhu *et al*. [13]	No	2016.07	2015.12	3	RCTs only	Change of LVEF	WMD	Fixed and Random effect	Study level
Ahn *et al*. [14]	No	2018.06	2017.11	11	RCTs only	Change of LVEF	RR and MD	Random effect	Study level
Khan *et al*. [15]	No	2018.05	2018.02	17	RCTs only	All-cause mortality	RR and MD	Random effect	Study level
Kheiri *et al*. [16]	Yes	2018.10	2018.02	7	RCTs only	HF hospitalization, all-cause mortality, serious adverse events	RR and WMD	Random effect	Study level
Elgendy *et al*. [17]	Yes	2018.09	2018.01	6	RCTs only	All-cause mortality	RR and SMD	Random effect	Study level
Briceño *et al*. [18]	No	2018.10	2018.02	7	RCTs only	All-cause mortality, change of LVEF	OR and SMD	Fixed and Random effect	Study level
Ma *et al*. [19]	No	2018.08	2018.02	7	RCTs only	All-cause mortality, HF hospitalization	RR	Random effect	Study level
Smer *et al*. [20]	No	2018.11	2018.02	6	RCTs only	LVEF, HF hospitalization, 6MWT, all-cause mortality	OR and MD	Random effect	Study level
Virk *et al*. [21]	No	2019.05	NA	6	RCTs only	Change of LVEF	RR and MD	Random effect	Study level
Turagam *et al*. [22]	No	2019.01	2017.09	6	RCTs only	All-cause mortality, HF hospitalization	RR	Random effect	Study level
Malik *et al*. [23]	No	2020.05	NA	17	RCTs only	All-cause mortality, HF hospitalization, change of LVEF	OR	Random effect	Study level
AlTurki *et al*. [24]	No	2019.01	2018.02	7	RCTs only	All-cause mortality	RR	Random effect	Study level
Moschonas *et al*. [25]	No	2018.09	2018.03	7	RCTs only	All-cause mortality	RR	Random effect	Study level
Agasthi *et al*. [26]	Yes	2019.04	2018.02	7	RCTs only	All‐cause mortality, HF hospitalization, AF recurrence	RR and MD	Random effect	Study level
Chen *et al*. [27]	No	2020.08	2019.04	11	RCTs only	All-cause mortality, re-hospitalization, stroke, thromboembolic events	OR and WMD	Random effect	Study level
Ruzieh *et al*. [28]	No	2019.01	2018.10	7	RCTs only	LVEF, MLHFQ scores, 6MWT, stroke, HF hospitalization, mortality	OR and MD	Random effect	Study level
Pan *et al*. [29]	No	2021.01	2019.09	6	RCTs only	All-cause mortality	RR	Random effect	Study level
Zhu *et al*. [30]	No	2021.12	2021.06	9	RCTs only	All-cause mortality, LVEF, 6MWT, MLHFQ scores	RR and MD	Random effect	Study level
Romero *et al*. [31]	No	2022.11	2022.04	8	RCTs only	All-cause mortality	RR	Fixed and Random effect	Study level
Yu *et al*. [32]	No	2022.09	2022.01	8	RCTs only	All-cause mortality	RR	Fixed and Random effect	Study level
Şaylık *et al*. [33]	No	2023.01	NA	10	RCTs only	All-cause mortality, LVEF, 6MWT, MLHFQ scores	RR and MD	Fixed and Random effect	Study level
Chang *et al*. [34]	No	2023.01	2021.06	7	RCTs only	HF hospitalization, all-cause mortality, serious adverse events	RR and MD	Random effect	Study level
Magnocavallo *et al*. [35]	No	2022.10	2022.05	9	RCTs only	Composite of all-cause mortality and HF hospitalization	RR	Fixed and Random effect	Study level
Khanra *et al*. [36]	No	2021.06	2020.10	12	RCTs only	All-cause mortality, change in QoL, AF recurrence and HF hospitalization	OR and MD	Random effect	Study level
Simader *et al*. [37]	Yes	2023.02	2022.03	8	RCTs only	All-cause mortality	RR	Fixed and Random effect	Study level
Sayed *et al*. [38]	Yes	2023.09	2022.06	9	RCTs only	All-cause mortality	RR	Fixed and Random effect	Study level
Casula *et al*. [39]	No	2023.04	2022.06	12	Mixed (RCTs + cohorts)	Mortality, hospitalization, LVEF, 6MWT	RR	Random effect	Study level
Lin *et al*. [40]	Yes	2023.03	2022.06	9	RCTs only	All-cause mortality, re-hospitalization, change of LVEF, AF recurrence	OR and MD	Random effect	Study level
Virk *et al*. [41]	No	2023.01	2022.03	9	RCTs only	All‐cause mortality, HF hospitalization, change of LVEF	RR and MD	Random effect	Study level
Lee *et al*. [42]	No	2023.05	2023.03	9	RCTs only	LVEF, 6MWT, HF questionnaire score, change of BNP, AF recurrence, HF hospitalization, all-cause mortality	OR	Random effect	Study level

Abbreviations: NA, not available; RCTs, randomized controlled trials; AF, atrial fibrillation; LVEF, left ventricular ejection fraction; HF, heart failure; 6MWT, 6-minute walk test; MLHFQ, Minnesota Living with Heart Failure questionnaire; QoL, quality of life; RR, relative risk; OR, odds ratio; MD, mean difference; WMD, weighted mean difference; SMD, standard mean difference; BNP, B-type natriuretic peptide.

### 3.2 Results of the Included Meta-Analyses

The results of included meta-analyses originated from 9 RCTs [43, 44, 45, 46, 47, 48, 49, 50, 51] and a subgroup analysis from one RCT—the CABANA trial [52], as listed in Table 3 (Ref. [9, 10, 11, 12, 13, 14, 15, 16, 17, 18, 19, 20, 21, 22, 23, 24, 25, 26, 27, 28, 29, 30, 31, 32, 33, 34, 35, 36, 37, 38, 39, 40, 41, 42]). Detailed information about these original studies can be found in **Supplementary Table 2**. Among the 9 RCTs, 8 conducted comparisons between AF ablation and standard drug therapy (comprising rate or rhythm control medications) in patients with HF [44, 45, 46, 47, 48, 49, 50, 51], while one contrasted AF ablation with rate control treatment utilizing atrioventricular junction ablation coupled with biventricular pacing, instead of drug-based therapies [43]. The CABANA trial aims to evaluate the superiority of catheter ablation over conventional medical therapy in enhancing outcomes among individuals with AF [52]. Notably, within this trial, 35% of patients presented with New York Heart Association class >II [52].

**Table 3.** **Map of original RCTs contained within each included meta-analysis**.

Authors of meta-analysis	PABA-CHF 2008	MacDonald *et al*. 2010	ARC-HF 2013	CAMTAF 2014	AATAC 2016	CAMERA-MRI 2017	CASTLE-AF 2018	AMICA 2019	CABANA HF-subgroup 2019/2021	RAFT-AF 2022
Dagres *et al*. [9]	√	√								
Vaidya *et al*. [10]	√	√	√	√						
Al Halabi *et al*. [11]	√	√	√	√						
Zhang *et al*. [12]	√	√	√	√						
Zhu *et al*. [13]		√	√	√						
Ahn *et al*. [14]	√	√	√	√		√				
Khan *et al*. [15]		√	√	√	√	√	√			
Kheiri *et al*. [16]	√	√	√	√	√	√	√			
Elgendy *et al*. [17]		√	√	√	√	√	√			
Briceño *et al*. [18]	√	√	√	√	√	√	√			
Ma *et al*. [19]	√	√	√	√	√	√	√			
Smer *et al*. [20]		√	√	√	√	√	√			
Virk *et al*. [21]		√	√	√	√	√	√			
Turagam *et al*. [22]		√	√	√	√	√	√			
Malik *et al*. [23]	√	√	√	√	√	√	√			
AlTurki *et al*. [24]	√	√	√	√	√	√	√			
Moschonas *et al*. [25]	√	√	√	√	√	√	√			
Agasthi *et al*. [26]	√	√	√	√	√	√	√			
Chen *et al*. [27]		√	√	√	√	√	√		√	
Ruzieh *et al*. [28]	√	√	√	√	√	√	√			
Pan *et al*. [29]		√	√	√	√	√	√			
Zhu *et al*. [30]		√	√	√	√	√	√	√		
Romero *et al*. [31]			√	√	√	√	√	√	√	√
Yu *et al*. [32]		√	√	√	√	√	√	√	√	
Şaylık *et al*. [33]	√	√	√	√	√	√	√	√	√	√
Chang *et al*. [34]		√	√	√	√	√	√	√		
Magnocavallo *et al*. [35]		√	√	√	√	√	√	√	√	√
Khanra *et al*. [36]	√	√	√	√	√	√	√	√		
Simader *et al*. [37]		√	√	√	√	√	√	√		√
Sayed *et al*. [38]		√	√	√	√	√	√	√	√	√
Casula *et al*. [39]		√	√	√	√	√	√	√	√	√
Lin *et al*. [40]		√	√	√	√	√	√	√	√	√
Virk *et al*. [41]	√	√	√	√	√	√	√	√		√
Lee *et al*. [42]		√	√	√	√	√	√	√	√	√

Abbreviations: RCTs, randomized controlled trials.

**Fig. 2.**
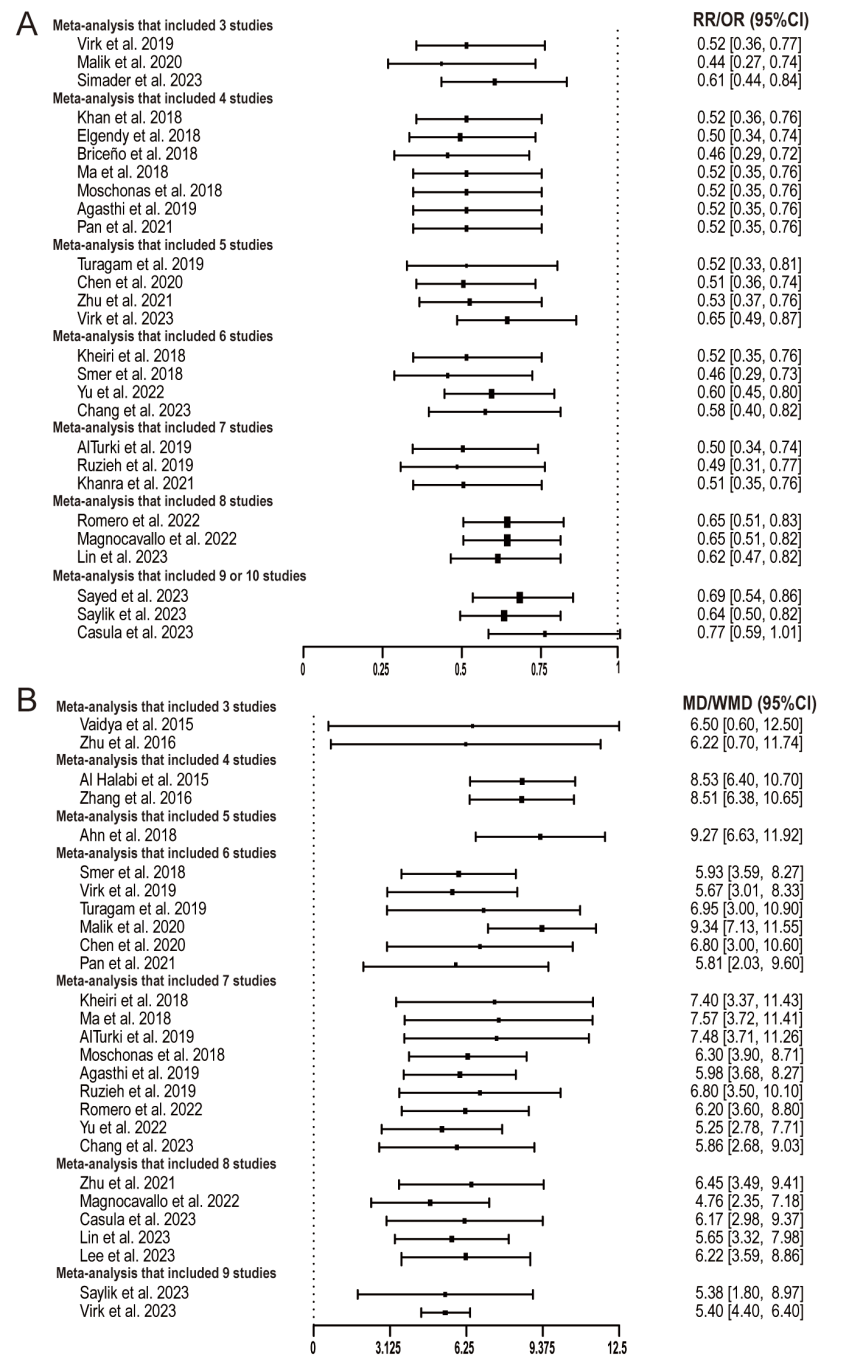
**Results of endpoint estimate by number of studies included in the meta-analyses ((A) all-cause mortality, (B) change of LVEF)**. LVEF, left ventricular ejection fraction; RR, relative risk; OR, odds ratio; MD, mean difference; WMD, weighted mean difference.

The findings across these meta-analyses underscore the burgeoning evidence supporting the advantages of catheter ablation for AF in patients with HF. This emphasizes the consistency across clinical trials favoring catheter ablation. Focusing on the critical hard endpoint, the majority of articles align in their conclusions, demonstrating a notable 40–50% decline in all-cause mortality among HF patients undergoing catheter ablation (Fig. 2A). Similarly, concerning the primary structural endpoint—the change of LVEF—most meta-analysis outcomes indicate that catheter ablation, in comparison to other treatments like drug therapy, can elevate LVEF by approximately 5–9% (Fig. 2B).

### 3.3 Citations and Cited Analysis

Previous research has indicated a tendency in meta-analyses to neglect prior systematic reviews and meta-analyses within the same topic [8]. In our study, there was a marked improvement compared to prior reports, with 79.4% of preceding meta-analyses being cited, 64.7% being described, and 50% of the outcomes being discussed within these included meta-analyses.

Since the initial meta-analysis was published in 2011, our search for clinical guidelines commenced from the subsequent year, 2012. Within this span, we identified 9 out of the 34 meta-analyses cited by ESC and ACC/AHA guidelines, garnering a total of 12 citations (**Supplementary Table 3**). Notably, two meta-analyses [11, 27] received citations from 2 and 3 guidelines, respectively.

## 4. Discussion

In this study, we evaluated the existing redundancy in meta-analyses pertaining to AF ablation among patients with HF. Our findings indicate a substantial surplus in the publication count of meta-analyses, surpassing the number of primary studies in this domain. Specifically, 34 meta-analyses can be generated based on 10 RCTs, but only 9 meta-analyses are cited by existing renowned guidelines. Simultaneously, there is a notable need for improvement in the frequency of citing, elaborating, and discussing on previous meta-analyses.

The management of patients with AF and HF poses significant challenges. Catheter ablation emerges as a beacon of hope for this particular patient cohort. As indicated by the meta-analyses’ findings included in our study, AF ablation among individuals with HF plays a pivotal role in enhancing quality of life, curbing HF hospitalizations, and mitigating mortality. In the latest 2023 ACC/AHA/ACCP/HRS guidelines on AF [53], catheter ablation has received a grade IA recommendation for AF and HF with reduced EF (HFrEF) patients who undergo guideline-directed medical therapy and with reasonable expectation of procedural benefit.

Setting aside these redundant meta-analyses, significant avenues remain unexplored within this domain. Firstly, existing RCT studies solely focus on HFrEF, lacking RCT evidence demonstrating the effectiveness of AF catheter ablation in patients with HF with preserved EF (HFpEF), despite numerous observational studies [54] and multiple published meta-analyses [55, 56]. Secondly, the outcomes attributing the reduction in mortality and hospitalization to catheter ablation in HFrEF patients were derived from a relatively limited number of events [57, 58], rendering definitive conclusions elusive. Consequently, further RCTs with expanded sample sizes are imperative. Consideration of conducting a Trial Sequential Analysis might aid in assessing whether anticipated information based on estimates can be attained, particularly in primary hard endpoints like mortality. Moreover, the comparative effectiveness of catheter ablation against alternative treatment options (such as assist devices and heart transplantation) in patients with severely reduced LVEF (<25%) remains uncertain. Additionally, the meta-analyses included in our study were all conducted at the study level, necessitating a patient-level meta-analysis for a comprehensive understanding.

### 4.1 Mass Production of Redundant Meta-Analyses

Indeed, the scientific community has acknowledged the issue of redundancy for over a decade [59, 60]. As stated at the outset of this article, there’s merit in moderately revisiting and updating meta-analyses. This practice enhances the capacity to unveil potentially meaningful outcomes by amalgamating individual studies. Moreover, it aids in pinpointing voids and methodological flaws within existing medical and public health literature, identifies potential sources of heterogeneity among studies, and lays the groundwork for critical future research avenues. However, the current landscape is witnessing an inundation of redundant meta-analyses, with a substantial proportion offering little or no added value.

Apart from concerns revolving around redundancy, there exist substantial apprehensions regarding methodology and quality within this domain. A case in point is highlighted by Milton Packer [58], who conducted a methodological assessment encompassing 14 meta-analyses on this topic. His findings revealed numerous errors committed by meta-analysis authors in the acquisition, extraction, and analysis of outcome data from the original RCTs. These errors could potentially jeopardize the reliability of the conclusions drawn from the meta-analyses. In a recent meta-analysis included in this present study, an inclusion criterion was purportedly limited to RCTs [30]. However, the inclusion of cohort studies within this meta-analysis has raised considerable concerns regarding its overall quality and adherence to specified criteria.

The ramifications of redundant meta-analyses are substantial. Firstly, they represent a significant misallocation of resources, consuming valuable human capital from researchers, reviewers, journals, and editors. This misdirection of efforts squanders energy and resources that could otherwise be channeled into more productive avenues. Secondly, there is a concerning possibility that these redundant meta-analyses may rely on or even plagiarize previous meta-analyses, casting doubts on their quality and originality. Such practices compromise the credibility of these articles. Additionally, the proliferation of numerous meta-analyses on the same topic contributes to information overload, potentially overshadowing more pertinent data. The absence of comparative analyses between these meta-analyses exacerbates this issue. Moreover, the inundation of “homogeneous” papers could impede the emergence of innovative ideas and inadvertently delay the publication of other exceptional articles [61]. New research papers harboring potentially groundbreaking concepts might encounter hurdles in publication, resulting in low visibility and citations. This conundrum of low visibility inhibits their dissemination and recognition within the academic sphere.

### 4.2 Potential Reasons

The proliferation of redundant meta-analyses stems from various factors. Firstly, heightened enthusiasm within the scientific community for certain topics contributes to the swift release of meta-analyses summarizing RCT studies following major study outcomes. This fervor is evident in the dual publication peaks observed in our study. Absolutely, the process of article publication involves several stages, including submission, review, and eventual publication, resulting in an inherent time delay. This inherent lag makes it challenging to entirely eliminate redundancy in published literature.

For authors, meta-analysis serves as a secondary analysis built upon original research. Advancements in statistical software, coding accessibility, modeling aids, and artificial intelligence tools have streamlined literature retrieval, data collection, and article composition, rendering meta-analysis implementation more standardized and approachable. Moreover, this trend may also be attributed to the mounting research pressure faced by scientists and clinicians. Research has previously highlighted that the escalating scientific research pressures encountered by young Chinese doctors have led to a rapid surge in published articles, potentially yielding redundant outputs [2, 62, 63].

Moreover, journals often accord prominence to meta-analyses due to their position at the apex of the evidence-level hierarchy. These studies are more prone to garner citations and might even be referenced within guidelines, contributing positively to the journal’s impact factor. Consequently, editors may exhibit a preference for publishing such articles owing to their potential to enhance the journal’s influence and visibility within the academic community [64].

### 4.3 Strategies for Improvement

Improvement measures to curtail the proliferation of redundant meta-analyses have been proposed, such as advocating for prospective registration via international agreement registries. However, these measures have proven insufficient in stemming the rising tide of redundant meta-analyses. In light of this, we propose the following suggestions.

Establishing a standardized meta-analysis production process is crucial. Requiring prospective registration for all meta-analyses, akin to the mandate for RCTs, would enhance transparency and help preempt redundancy. While the PRISMA 2020 checklist has significantly improved the quality and reporting standards for meta-analyses, its impact on resolving redundancy has been limited [7]. To address this, we propose that major entities expand upon the PRISMA checklist tailored to their specific needs. This extended checklist would provide detailed guidance, contextualized to the unique requirements of each field or domain. By offering a comprehensive framework, authors can gain deeper insights into the checklist’s nuances and apply it more effectively, thereby potentially reducing redundancy in meta-analyses.

Absolutely, journals and editors play a pivotal role in minimizing redundancy in meta-analyses. Encouraging additional instructions from authors during the submission of meta-analysis articles could significantly contribute to this effort. Many journals require authors to provide highlights, but it is very easy to find so-called highlights, and authors are likely to ignore published meta-analyses on this topic. Implementing formats like those seen in the Lancet series, which require authors to provide information on the Evidence before the study, the Added value of the current study, and the Implications based on all available evidence, can be more effective than traditional highlight sections. Similarly, initiatives like the JAMA network open editors’ requirement for contributors to include a cover letter explaining the freshness of the submitted meta-analysis, detailing previous meta-analyses conducted in the past five years on the topic, and demonstrating consistency or comparative analyses with prior studies, are instrumental in mitigating redundancy [65]. These detailed and stringent requirements compel authors to critically evaluate existing literature, thereby enhancing the value and uniqueness of the submitted meta-analyses. Accelerating the publication timeline for accepted meta-analyses enables swift dissemination of novel findings, potentially deterring overlapping meta-analyses and enhancing the impact of new contributions.

Peer review also plays a critical role in reducing redundancy in meta-analyses. Reviewers are recommended to intensify their scrutiny of potential redundancy during the peer review process by simply searching for recently published similar meta-analyses. Additionally, they should check whether the authors have cited and discussed previous meta-analyses in their article. This ensures that the proposed meta-analysis genuinely contributes novel insights or methods to the existing knowledge base.

For authors of RCTs, we advocate for the upload of de-identified raw data or providing more efficient methods for data utilization. Meta-analysis authors are also encouraged to conduct comparative analyses with existing meta-analyses, aiming to pinpoint gaps, inconsistencies, or duplications in the current literature before commencing new studies.

To sum up, enhancing communication among trialists, implementing targeted quality controls at the editor and reviewer levels, promoting living systematic reviews, prospective registration of systematic review protocols, and updating the PRISMA checklist to address redundancy and selective reporting bias. These measures, as suggested by Riaz *et al*. [66], are pivotal steps toward mitigating redundant publications in meta-analyses.

## 5. Limitation

Several limitations warrant acknowledgment in this study. Firstly, inherent limitations within the search databases and temporal constraints might have resulted in the omission of additional published literature. Secondly, our study did not entail an evaluation of the quality of the included meta-analyses, as this aspect lay beyond the scope of our focus. Thirdly, a comparative analysis of the specific differences among these meta-analyses was not conducted. Future investigations are warranted to explore and delineate the variations among meta-analyses concerning the same topic, allowing for a more comprehensive resolution of this issue.

## 6. Conclusions

Currently, there is a massive production of unnecessary meta-analyses on catheter ablation in AF and HF. The prevalence of redundancy in meta-analysis has emerged as a pressing concern, demanding more robust and effective measures for mitigation.

## Availability of Data and Materials

All data used in the current study were included in the manuscript and supplementary materials.

## Supplementary Material

Supplementary material associated with this article can be found, in the online version, at https://doi.org/10.31083/j.rcm2511418.
