# Supplementary material for: Assessment of Redundant Meta-Analyses on Catheter Ablation of Atrial Fibrillation in Patients with Heart Failure
Source: Rev Cardiovasc Med. 2024 Nov 21;25(11):418. doi: 10.31083/j.rcm2511418 (PMC11607499; doi:10.31083/j.rcm2511418)
Supplement: Supplementary file 1 [file 2153-8174-25-11-418-s1.zip › Supplementary Material-V1.docx]

**Assessment of redundant meta-analyses on catheter ablation of atrial fibrillation in patients with heart failure**

**Supplementary files:**

Supplementary Table 1. Search strategy and results.

Supplementary Table 2. Study characteristics of 10 RCTs on catheter ablation in patients with AF and HF.

Supplementary Table 3. The recommendations of most-impact guidelines on AF catheter ablation in patients with HF.

**Supplementary Table 1. Search strategy and results**

| **Database** | **Search queries** | **Terms** | **Results (Dec 25, 2023)** |
| --- | --- | --- | --- |
| **PubMed** | #1 | “atrial fibrillation”[tiab] OR “Atrial Fibrillation”[MeSH Terms] | 108,795 |
|  | #2 | “heart failure”[tiab] OR “cardiac failure”[tiab] OR “myocardial failure”[tiab] OR “heart decompensation”[tiab] OR “decompensation, heart”[tiab] OR “left ventricular dysfunction”[tiab] OR “Heart Failure”[Mesh] | 278,650 |
|  | #3 | “catheter ablation”[tiab] OR “ablation therapy”[tiab] OR “radiofrequency ablation”[tiab] OR “cryoablation”[tiab] OR “cryoballoon ablation”[tiab] | 41,687 |
|  | #4 | “meta-analysis”[tiab] OR “pooling analysis”[tiab] OR “integrated analysis”[tiab] OR “synthesis analysis”[tiab] OR “Meta-Analysis”[Publication Type] | 288,023 |
|  | #5 | #1 and #2 and #3 and #4 | 70 |
| **Embase** | #1 | 'atrial fibrillation':ti,ab,kw | 170,674 |
|  | #2 | 'heart failure':ti,ab,kw OR 'cardiac failure':ti,ab,kw OR 'myocardial failure':ti,ab,kw OR 'heart decompensation':ti,ab,kw OR 'left ventricular dysfunction':ti,ab,kw | 402,679 |
|  | #3 | 'catheter ablation':ti,ab,kw OR 'ablation therapy':ti,ab,kw OR 'radiofrequency ablation':ti,ab,kw OR 'cryoablation':ti,ab,kw OR 'cryoballoon ablation':ti,ab,kw | 69,879 |
|  | #4 | 'meta analysis':ti,ab,kw OR 'pooling analysis':ti,ab,kw OR 'integrated analysis':ti,ab,kw OR 'synthesis analysis':ti,ab,kw | 328,349 |
|  | #5 | #1 AND #2 AND #3 AND #4 | 105 |
| **Web of Science** | #1 | TS=“atrial fibrillation” | 110,567 |
|  | #2 | TS=“heart failure” OR TS=“cardiac failure” OR TS=“myocardial failure” OR TS=“heart decompensation” OR TS=“left ventricular dysfunction” | 283,657 |
|  | #3 | TS=“catheter ablation” OR TS=“ablation therapy” OR TS=“radiofrequency ablation” OR TS=“cryoablation” OR TS=“cryoballoon ablation” | 54,533 |
|  | #4 | TS=“meta analysis” OR TS=“pooling analysis” OR TS=“integrated analysis” OR TS=“synthesis analysis” | 287,780 |
|  | #5 | #1 and #2 and #3 and #4 | 105 |

**Supplementary Table 2. Study characteristics of 10 RCTs on catheter ablation in patients with AF and HF**

| **Trial Name** | **Year of publication** | **Region** | **Number of study population** | **Age (y)** | **Male (%)** | **Follow-up** | **Primary Outcome** | **Outcome Measure** | **DOI** |
| --- | --- | --- | --- | --- | --- | --- | --- | --- | --- |
| PABA-CHF | 2008 | USA | 81 | 60±8* | 95* | 6 months | Composite of LVEF, distance on the 6-minute walk test, or MLWHF score | LVEF (35±9% vs. 28±6%); 6-minute walking distance (340±49 m vs. 297±36 m); MLWHF score (60±8 vs. 82±14) | 10.1056/NEJMoa0708234 |
| MacDonald et al. | 2010 | UK | 41 | 62.3±6.7* | 77* | 6 months | Change of LVEF | 4.56±11.1% vs. 2.86±6.7% | 10.1136/hrt.2010.207340 |
| ARC-HF | 2013 | UK | 52 | 63±9 | 81* | 12 months | Change in peak oxygen consumption | MD 3.07 (95%CI 0.56-5.59) | 10.1016/j.jacc.2013.01.069 |
| CAMTAF | 2014 | UK | 50 | 55±12* | 96 | 6 months | LVEF | 40±12% vs. 31±13% | 10.1161/CIRCEP.113.000806 |
| AATAC | 2016 | USA | 203 | 62±10* | 75* | 24 months | AF recurrence | 70% (95% CI 60%-78%) vs. 34% (95% CI 25%-44%) | 10.1161/CIRCULATIONAHA.115.019406 |
| CAMERA-MRI | 2017 | Australia | 66 | 59±11* | 94* | 6 months | Change of LVEF | 18±13% vs. 4.4±13% | 10.1016/j.jacc.2017.08.041 |
| CASTLE-AF | 2018 | Europe, Australia, USA | 363 | 64 | 85.5 | 37.8 months | Composite of death and HF hospitalization | HR 0.62 (95% CI 0.43-0.87) | 10.1056/NEJMoa1707855 |
| AMICA | 2019 | Europe | 140 | 65±8 | 90 | 12 months | Absolute increase in LVEF | 8.8% (95% CI 5.8%-11.9%) vs. 7.3% (95% CI 4.3%-10.3%) | 10.1161/CIRCEP.119.007731 |
| CABANA HF subgroup | 2021 | USA, Australia, Canada, China, Czechia, Germany, Italy, Korea, Russian Federation, UK | 778 | 68 (62, 73) | 55.7 | 48.5 months | Composite of death, disabling stroke, serious bleeding, or cardiac arrest | HR 0.64 (95% CI 0.41-0.99) | 10.1161/CIRCULATIONAHA.120.050991 |
| RAFT-AF | 2022 | Brazil, Canada, Sweden, Taiwan | 411 | 65.9±8.6* | 73.4* | 37.4 months | Composite of all-cause mortality and HF events | HR 0.71 (95% CI 0.49-1.03) | 10.1161/CIRCULATIONAHA.121.057095 |

Note: *Data in the catheter ablation group.

**Supplementary Table 3. The recommendations of most-impact guidelines on AF catheter ablation in patients with HF**

| **Guideline** | **Recommendations** | **Class of recommendation** | **Level of evidence** | **Meta-analyses cited*** |
| --- | --- | --- | --- | --- |
| 2012 ESC guidelines on HF | The role of catheter ablation as a rhythm control strategy in HF is at present uncertain. | N/A | N/A | No |
| 2012 update of ESC guidelines on AF | In patients who suffer from symptomatic AF recurrences on amiodarone therapy, catheter ablation remains as the sole choice for escalated rhythm control therapy. | N/A | N/A | No |
| 2013 ACCF/AHA guidelines on HF | Not mentioned. | N/A | N/A | No |
| 2014 AHA/ACC/HRS guidelines on AF | Not mentioned. | N/A | N/A | No |
| 2016 ESC/EACTS guidelines on AF | AF ablation should be considered in symptomatic patients with AF and heart failure with reduced ejection fraction to improve symptoms and cardiac function when tachycardiomyopathy is suspected. | IIa | C | 26258174 |
| 2016 ESC guidelines on HF | AF ablation may be considered in order to restore sinus rhythm to improve symptoms in patients with persisting symptoms and/or signs of HF, despite OMT and adequate control of ventricular rate, to improve clinical/symptomatic status. | IIb | B | No |
| 2017ACC/AHA/HFSA update of guidelines on HF | Not mentioned. | N/A | N/A | No |
| 2019AHA/ACC/HRS update of guidelines on AF | AF catheter ablation may be reasonable in selected patients with symptomatic AF and HF with reduced left ventricular (LV) ejection fraction (HFrEF) to potentially lower mortality rate and reduce hospitalization for HF. | IIb | B | 26258174 |
| 2020 ESC/EACTS guidelines on AF | AF catheter ablation is recommended to reverse LV dysfunction in AF patients when tachycardia-induced cardiomyopathy is highly probable, independent of their symptom status. | I | B | 22041335 |
|  | AF catheter ablation should be considered in selected AF patients with HF with reduced LVEF to improve survival and reduce HF hospitalization. | IIa | B | 26258174,30066291,30103676,30001942,29798790 |
| 2021 ESC guidelines on HF | In cases of a clear association between paroxysmal or persistent AF and worsening of HF symptoms, which persist despite MT, catheter ablation should be considered for the prevention or treatment of AF. | IIa | B | No |
| 2022 ACC/AHA/HFSA guidelines on HF | For patients with HF and symptoms caused by AF, AF ablation is reasonable to improve symptoms and QOL. | IIa | B | 31298266 |
| 2023 ACC/AHA/ACCP/HRS guidelines on AF | In appropriate patients with AF and HFrEF who are on GDMT, and with reasonable expectation of procedural benefit, catheter ablation is beneficial to improve symptoms, QOL, ventricular function, and cardiovascular outcomes. | I | A | 31298266,33628087,30583296 |
|  | In appropriate patients with symptomatic AF and HFpEF with reasonable expectation of benefit, catheter ablation can be useful to improve symptoms and improve QOL. | IIa | B | No |

Note: *PubMed ID.
